# Acute Myocardial Infarction During the COVID-19 Pandemic: An Update on Clinical Characteristics and Outcomes

**DOI:** 10.3389/fcvm.2021.648290

**Published:** 2021-12-23

**Authors:** Olga Toscano, Nicola Cosentino, Jeness Campodonico, Antonio L. Bartorelli, Giancarlo Marenzi

**Affiliations:** ^1^Centro Cardiologico Monzino, I.R.C.C.S., Milan, Italy; ^2^Department of Clinical Sciences and Community Health, Cardiovascular Section, University of Milan, Milan, Italy; ^3^Department of Biomedical and Clinical Sciences “Luigi Sacco,” University of Milan, Milan, Italy

**Keywords:** acute myocardial infarction, COVID-19, epidemiology, clinical characteristic, outcome

## Abstract

The outbreak of coronavirus disease 2019 (COVID-19) has rapidly become a worldwide pandemic. On top of respiratory complications, COVID-19 is associated with major direct and indirect cardiovascular consequences, with the latter probably being even more relevant, especially in the setting of time-dependent cardiovascular emergencies. A growing amount of data suggests a dramatic decline in hospital admissions for acute myocardial infarction (AMI) worldwide during the COVID-19 pandemic, mostly since patients did not activate emergency medical systems because hospitals were perceived as dangerous places regarding the infection risk. Moreover, during the COVID-19 pandemic, patients with AMI had a significantly higher in-hospital mortality compared to those admitted before COVID-19, potentially due to late arrival to the hospital. Finally, no consensus has been reached regarding the most adequate healthcare management pathway for AMI and shared guidance on how to handle patients with AMI during the pandemic is still needed. In this review, we will provide an update on epidemiology, clinical characteristics, and outcomes of patients with AMI during the COVID-19 pandemic, with a special focus on its collateral cardiac impact.

## Introduction

Coronavirus disease 2019 (COVID-19), a novel viral respiratory illness due to severe acute respiratory syndrome coronavirus 2 (SARS-CoV-2), became a global pandemic in 2020 (1). As we continued to fight against the infectious disease and the rapid contagion of the virus, we understood that, besides being primarily a respiratory illness, COVID-19 has potential direct and indirect cardiac sequelae that were initially underestimated (2, 3). Indeed, several reports have described relevant cardiac complications in patients with COVID-19 with or without the prior cardiovascular disease (2, 3). Of note, even the latter patients are more likely to have an acute myocardial infarction (AMI), heart failure, and life-threatening arrhythmias due to a direct impact of SARS-CoV-2 infection on the cardiovascular system (4). However, the pandemic might have even had more severe indirect sequelae. In particular, despite all the great efforts made by the international health authorities and national governments to fight the infection, the patients with COVID-19 surge in demand for Intensive Care Unit (ICU) admission has been overwhelming (5, 6). As a consequence, also Intensive Cardiac Care Units (ICCU) have been dedicated to the treatment of patients with pneumonia and severe acute respiratory syndrome. Thus, the tremendous pressure exerted on the healthcare system by the viral pandemic compromised proven therapies for acute cardiovascular emergencies, such as AMI (7, 8). Another serious issue during the COVID-19 outbreak has been the reluctance of patients with chest pain to go to the hospital due to the fear of viral infection, even to the point of not seeking care at all or late in the course of AMI (9–11). These indirect effects of the pandemic have negatively affected the outcomes of patients with AMI, regardless of whether they were affected by SARS-CoV-2 infection or not.

In this review, we will provide an update on epidemiology, clinical characteristics, and outcomes of patients with AMI during the COVID-19 pandemic, with a special focus on its collateral impact on AMI. With the SARS-CoV-2 infection still not being under control, understanding and addressing the relationship between COVID-19 and AMI is critical if we want to prevent a further increase in mortality and a new heart failure pandemic wave.

## Possible Mechanisms Linking COVID-19 to AMI

Several mechanisms associated with COVID-19 may be involved in AMI. Type 1 AMI can be triggered in patients with COVID-19 by a pro-inflammatory state, which may promote destabilization of a coronary atherosclerotic plaque, a phenomenon already observed during influenza outbreaks (12). Notably, viral infections have been shown to activate inflammatory cells of the coronary plaque and to upregulate metalloproteinases and peptidases, which, in turn, may disrupt plaque cap exposing the highly thrombogenic core to the blood (13). Another potential mechanism is the mismatch between reduced oxygen supply and increased myocardial oxygen demand due to sympathetic system activation, tachycardia, hypotension, and hypoxemia in the setting of acute respiratory insufficiency, which may be responsible for Type 2 AMI (14). Moreover, other mechanisms related to specific features of SARS-CoV-2 infection have been advocated to explain AMI in patients with COVID-19. In particular, the endothelial and microvascular injuries induced by SARS-CoV-2 may further enhance inflammation, resulting in coronary vasospasm, thrombosis, and myocardial perfusion defects (15). Moreover, the low platelet count often described in patients with COVID-19 suggests an increased consumption due to great platelet activation and thrombus formation. Indeed, the cytokine storm associated with viral infection induces, together with the imbalance of endothelial function, significant activation of platelets, granulocytes, and microvesicles, which, in turn, produce tissue factors (16). Of note, it has also been demonstrated that plasma microvesicles-associated thrombin generation can still be present in patients with COVID-19 despite prophylactic anticoagulation (16).

Another possible mechanism implicated in the association between SARS-CoV-2 and AMI is the pro-inflammatory state. Since the association between infection and acute coronary atherothrombosis has been established for a variety of pathogens and sites of infection, it is likely that the causal agent and the host response could have a crucial role in eliciting an inflammatory pattern that may trigger AMI. Atherosclerotic plaques contain inflammatory cells that proliferate, secrete cytokines, and stimulate smooth muscle cells to form a fibrous cap. Thus, an inflammatory status generates circulating cytokines that may activate inflammatory cells in atherosclerotic plaques, enhancing plaque vulnerability and the possibility of its rupture, leading to coronary thrombosis (14).

Of note, there are multiple reports of microvascular involvement in different organs of patients with COVID-19, leading to ischemic stroke (17), deep vein thrombosis (18), pulmonary embolism (19), and arterial thrombotic events (20).

The COVID-19 has more far-reaching cardiovascular implications than the pathophysiological effects of the disease *per se*. In fact, all countries have developed containment strategies based on social distancing, and it is well-known that the lack of human relationships and reduced interaction with other people are major risk factors for cardiovascular mortality. A previous meta-analysis includes 181,000 subjects demonstrated that the risk for AMI increases by almost 30% in lonely and socially isolated people (21). The adult cohort studies reported initial evidence of a clinically meaningful increase in anxiety, depression, mental health disturbance, and disruption of well-being during the lockdown for SARS-CoV-2 spread containment, all of which have been associated with an increased AMI risk (22).

## Epidemiology of AMI During the COVID-19 Pandemic

In the early period of the pandemic, many healthcare workers noticed a reduction in hospital admissions for AMI. This finding was largely consistent across continents and, although initially based on self-reported perceptions (23), it was then supported by objective evidence from worldwide registries, suggesting a 25–40% decrease in AMI admissions during the outbreak (Table 1). Xiang et al. (24) looked into the China Chest Pain Center Database to evaluate the impact of the COVID-19 pandemic on ST-elevation myocardial infarction (STEMI) admission in the 4 weeks before and after January 24, 2020 (the start date of the COVID-19 outbreak in China). They found an approximately 25% drop in the weekly number of patients hospitalized for STEMI during the COVID-19 outbreak nationwide, and about a 60% drop in Hubei province. In a multicenter, observational survey, De Rosa et al. (25) collected data from 54 ICCU across Italy during 1-week period at the beginning of the COVID-19 outbreak. A halving in AMI admissions was registered during the 2020 week compared with the equivalent 2019 week. Because of deep regional variations in COVID-19 involvement in Italy, with the north being the most affected area, the country was divided into three macro-areas (north, central, and south Italy), and the authors still found a similar decline in AMI admissions among these macro-areas. Similarly, a Spanish report compared the activity of 81 ICCU a week before the pandemic with that of a week during the pandemic. The authors observed a significant reduction in ICCU activity mainly due to a marked decrease in STEMI hospitalization, with a concerning 40% decline in primary percutaneous coronary intervention (PCI) (26). Likewise, during the early phase (March 2020) of the COVID-19 pandemic, a 38% reduction of primary PCI activity was reported in nine high-volume catheterization laboratories of the United States (27). The same authors confirmed the marked reduction in interventional activity during April 2020 in a survey of 18 United States STEMI centers. Interestingly, the decline in hospital admissions for STEMI was seen in all geographic areas of the United States, irrespective of COVID-19 incidence, implementation of lockdown, and level of SARS-CoV-2 testing (35). Finally, another survey of more than 3,000 health professionals from 141 countries, endorsed by the European Society of Cardiology (ESC), showed an important decline in patients admitted to hospital for AMI during the pandemic (23). Notably, the responses received showed that 80% of health professionals felt that there had been a decrease in presentations, with the large majority of participants reporting at least a 40% reduction. Later on, nationwide analysis of acute coronary syndrome admissions conducted in other geographical areas that had lockdown restrictions, such as England (28), France (29), Greece (30), and California (31), showed the same concerning trend. Finally, Mohammad et al. (32) recorded a nationwide significant decline in AMI presentation during the COVID-19 pandemic as compared to the corresponding period of previous years (2015–2019) also in Sweden, that, unlike other countries, did not impose mandatory lockdown.

**Table 1:** Characteristics of the studies investigating the admission rate for acute myocardial infarction during the COVID-19 pandemic.

**First author [Ref#]**	**AMI type**	**Country**	**COVID-19 period considered**	**Patients (*n*)**	**Control period considered**	**Patients (*n*)**	**Percent change in AMI admissions**
Xiang et al. (24)	STEMI	China	27 Dec 2019–23 Jan 2020	15,729	24 Jan−20 Feb 2020	11,598	−26%
De Rosa et al. (25)	STEMI/NSTEMI	Italy	12 Mar−19 Mar 2020	319	12 Mar−19 Mar 2019	618	−48%
Rodriguez-Leor et al. (26)	STEMI	Spain	16 Mar−22 Mar 2020	260	24 Jan−1 Mar 2020	433	−40%
Garcia et al. (27)	STEMI	United States	1 Mar−31 Mar 2020	138	1 Jan 2019–29 Feb 2020	>180/month	−38%
Mafham et al. (28)	STEMI/NSTEMI	England	1 Jan−24 May 2020	1,813/week	1 Jan−31 Dec 2019	3,017/week	−40%
Mesnier et al. (29)	STEMI/NSTEMI	France	16 Mar−12 Apr 2020	481	17 Feb−15 Mar 2020	686	−30%
Papafaklis et al. (30)	ACS	Greece	2 Mar−12 Apr 2020	771	2 Mar−12 Apr 2019	1,077	−38%
Solomon et al. (31)	STEMI/NSTEMI	United States	4 Mar−14 Apr 2020	516	4 Mar−14 Apr 2019	735	−30%
Mohammad et al. (32)	STEMI/NSTEMI	Sweden	1 Mar−7 May 2020	36/day	1 Mar−7 May 2015-2019	45/day	−20%
Gluckman et al. (33)	STEMI/NSTEMI	United States	23 Feb−28 Mar 2020	860	30 Dec 2018–22 Feb 2020	-	−19%
Wilson et al. (34)	STEMI	United Kingdom	19 Feb−8 Apr 2020	388	19 Feb−8 Apr 2017-2019	-	−51%

*ACS, acute coronary syndrome; NSTEMI, non-ST elevation myocardial infarction; STEMI, ST-elevation myocardial infarction*.

Several causes may explain the reduction in AMI admissions, such as patient reluctance to go to the hospital for fear of being exposed to SARS-CoV-2 or to overload an already strained health service, and delay in response of a congested ambulance and emergency service. The hypothesis that patients avoided access to the emergency departments because of contagion fear is supported by the lack of significant differences in AMI admission among the Italian macro-areas assessed by De Rosa et al. (25), despite great discrepancies in COVID-19 spread across the country. Swedish results are consistent with this data, showing that AMI admissions declined, when compared to previous years, even when areas as the COVID-19 hotspot in Stockholm were excluded from the analysis (32). However, we cannot exclude that some patients with AMI, who experienced dyspnea, only misjudged the symptom as COVID-19 related and chose to remain at home, without seeking care. Furthermore, social distancing and improved hygiene might have attenuated the spreading of influenza, a widely recognized AMI trigger (12). Another suggested hypothesis is the arrangement of healthcare resources during the pandemic with deferral of less urgent cases. In line with this theory, De Rosa et al. (25) showed less reduction in hospitalization for STEMI compared with non-STEMI (NSTEMI), a finding also reported by Mesnier et al. (29) in a French registry. Finally, it has been suggested that the widespread working from home, especially after the implementation of lockdown measures, may have contributed to decrease stress-induced AMI. However, as indicated both by the United States and English data, the drop in AMI admissions preceded the start of the lockdown by 2 weeks and 1 month, respectively, thus suggesting that the above-mentioned condition might have played a minor role (36).

The reluctance of patients with AMI to go to the hospital due to the fear of being exposed to SARS-CoV-2 is also suggested by the significant increase in out-of-hospital cardiac arrests (OHCA) reported during the COVID-19 outbreak. This association was first observed in New York City, particularly, from March 30 to April 5, 2020. Indeed, during this period, there were 1,990 OHCA calls, a rate four times higher than that reported during the same time interval a year before (37). The dramatic fact was that this was associated with an eight times higher mortality. Later, Baldi et al. (38) compared the number of OHCA occurring in four Italian provinces with the highest rate of COVID-19 cases in the first 40 days of the outbreak to the same period of the previous year. The analysis showed a strong association between the cumulative incidence of OHCA and COVID-19 disease. Furthermore, they observed that the 60% increase in OHCA in 2020 compared to the same period in 2019 paralleled the time course of the COVID-19 outbreak. A similar significant increase of OHCA during the pandemic was also observed in an American cross-sectional study (37). Of note, this study reported that patients with OHCA presented more frequently with asystole and pulseless electrical activity than ventricular fibrillation or ventricular tachycardia. In addition, the rate of spontaneous circulation recovery was significantly lower during the COVID-19 period than in 2019. However, none of the above studies reported data regarding AMI diagnosis or history of coronary artery disease in the patients included in the analyses. One more piece of information comes from the study of Rashid et al. (39) who showed an almost double incidence of OHCA during a defined COVID-19 period compared to a pre-COVID-19 period in a cohort of patients hospitalized with AMI, substantiating the concerns that reduced AMI admissions may have resulted in an increased risk of OHCA.

## Clinical Characteristics and Outcomes of Patients With AMI During COVID-19

To date, evidence concerning the clinical characteristics and in-hospital outcomes of AMI patients during the COVID-19 pandemic is limited, and it mainly derives from single-center experiences, with most studies reporting partial details on patient baseline risk, comorbidities, and clinical outcomes (Table 2) (28, 41).

**Table 2:** Characteristics of the studies investigating the clinical impact of the COVID-19 pandemic on patients with acute myocardial infarction.

**First author [Ref#]**	**Study population**	**Age** **(years)**	**Gender (males)**	**Mortality pandemic period**	**Mortality pre-pandemic period**	**AMI complications pandemic period**	**AMI complications pre-pandemic period**
Cosentino et al. (10)	STEMI	64 ± 12	83%	19%	5%	CS 21%	CS 9%
Xiang et al. (24)	STEMI	63 ± 13	75%	5%	4%	AHF 14%	AHF 13%
De Rosa et al. (25)	STEMI/NSTEMI	68 ± 9	76%	10%	3%	16%^*^	7%^*^
Mesnier et al. (29)	STEMI/NSTEMI	65 ± 13	74%	5%	3%	Killip III–IV 9%	Killip III–IV 8%
Papafaklis et al. (30)	ACS	64 (56–74)	79%	3.3%	2.7%	CS 6.1%	CS 5.2%
Mohammad et al. (32)	STEMI/NSTEMI	70 (61–77)	67%	12%	6%	Killip III–IV 2.4%	Killip III–IV 2.4%
Gluckman et al. (33)	STEMI/NSTEMI	67 ± 13	68%	5%	5%	-	-
Carugo et al. (40)	STEMI/NSTEMI	69 (58–77)	77%	9%	-	CS 8%	-
Wilson et al. (34)	STEMI	63	68%	15%	11%	CS 18%	CS 19%

*ACS, acute coronary syndrome; AHF, acute heart failure; AMI, acute myocardial infarction; CS, cardiogenic shock; NSTEMI, non-ST elevation myocardial infarction; STEMI, ST-elevation myocardial infarction*.

* *Cardiogenic shock, life-threatening arrhythmias, cardiac rupture/ventricular septal defect, or severe functional mitral regurgitation*.

Clinical observations made in England about the characteristics of patients with AMI during the pandemic lockdown showed that they were younger, less frequently diabetics, and less likely to have a history of prior cerebrovascular disease, as compared to those admitted during the previous year (28). Similar data were found in a retrospective cross-sectional study analyzing patients with STEMI and NSTEMI admitted between December 30, 2018 and May 16, 2020 in 49 hospitals in the Providence St. Joseph Health (PSJH) system that spreads across Alaska, California, Montana, Oregon, Texas, and Washington. This study showed that patients hospitalized during a defined COVID-19 period were younger and more likely to be Asian or Native American than the ones hospitalized before (33). On the other hand, a Swedish registry reported no difference (both at a nationwide level and in Stockholm) in age, gender, and comorbidities except for lower rates of prior AMI and coronary artery bypass grafting in patients with AMI during the pandemic (32). In line with the Swedish observation, both a French registry by Mesnier et al. (29) and a single-center German study by Primessnig et al. (42) showed that age, gender, and prevalence of risk factors did not differ between the pre-pandemic and pandemic period in patients with AMI. In northern California, patients presenting with AMI during the COVID-19 outbreak were less likely to have a history of coronary artery disease compared to patients presenting during the pre-COVID-19 period (31). However, there was not any difference in terms of demographic characteristics and comorbidities in the two periods.

An observation common to studies was that during the pandemic a higher percentage of patients were admitted with STEMI as compared to NSTEMI (25, 28, 40). Indeed, a large database of 99 English hospitals showed that, on average, hospitalization for NSTEMI was reduced by 50% and by 25% for STEMI (28). Likewise, a multicenter observational survey examining 319 consecutive patients with AMI in the week with the highest peak of COVID-19 spread in Italy reported a decrease in hospital admission by 27% for STEMI and by 65% for NSTEMI (25). The greater reduction in NSTEMI admissions might have several explanations. There is the chance that patients with NSTEMI did not seek medical help because their symptoms were less frequently characterized by precordial pain or chest discomfort, thus increasing their reluctance to expose themselves to the in-hospital risk of COVID-19 infection. In line with this hypothesis, data from the Lombardy region in Italy showed that, during the COVID-19 pandemic, patients with AMI presented more frequently with dyspnea and atypical symptoms (40). In addition, an association between increasing age and pre-existing comorbidities and a poorer outcome following COVID-19 infection was largely emphasized by the media at the start of the pandemic, affecting the choice of some patients with NSTEMI to remain at home, since they considered themselves at high risk in case of infection due of their older age and concomitant illnesses.

An important observation made during the COVID-19 pandemic was that patients with STEMI had greater enzymatic infarct size, as assessed by the peak of troponin or creatine kinase levels (42, 43), lower left ventricular ejection fraction (34, 42), higher intracoronary thrombotic burden (44), and, therefore, more frequent in-hospital complications. Indeed, a higher rate of cardiogenic shock, need for inotropic and mechanical hemodynamic support, and an increased incidence of life-threatening ventricular arrhythmias after successful revascularization of the culprit artery were found in patients with AMI admitted during the COVID-19 pandemic, with higher early mortality (10, 11, 25, 33, 42, 45). In particular, De Rosa et al. (25) found that in-hospital mortality for STEMI increased to 14% during the pandemic as compared to a 4% rate in the same period of 2019. In their work, De Rosa et al. found that major complications (cardiogenic shock, life-threatening arrhythmias, cardiac rupture, and severe mitral regurgitation) were also increased from 10% of the previous year to 19%. Moreover, a study carried out in London found that not only higher in-hospital mortality in patients with STEMI but also a raised length of stay during the peak of the pandemic (1 march to 30 April 2020) compared to those observed during the corresponding 2019 period (46).

**Figure 1:**
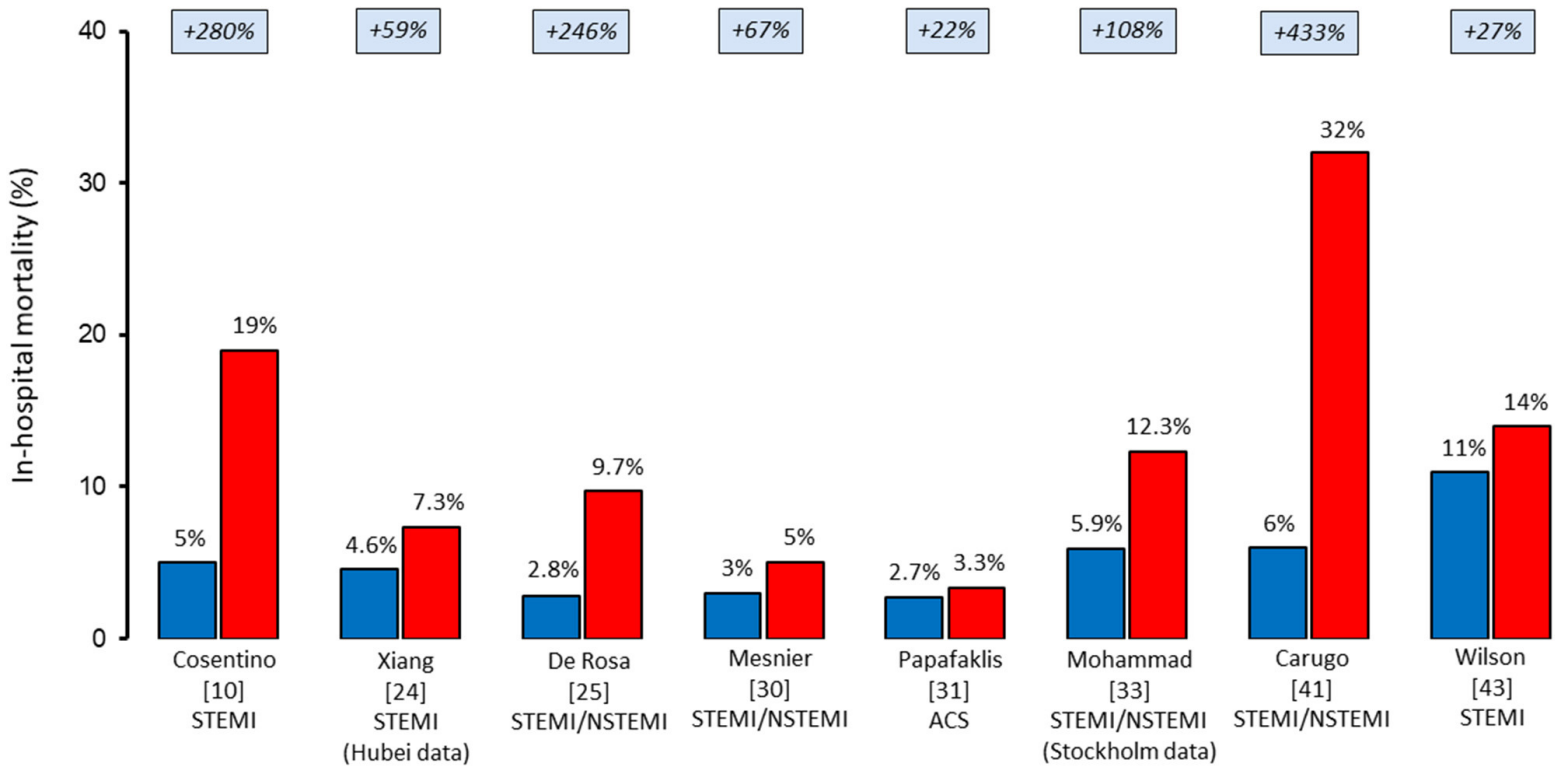
In-hospital mortality rates of patients hospitalized with acute myocardial infarction before (blue) and during (red) the COVID-19 pandemic. ACS, acute coronary syndrome; NSTEMI, non-ST-elevation myocardial infarction; STEMI, ST-elevation myocardial infarction.

The pandemic caused significant disruption in AMI workflow, with a 39% increase in time from symptom onset to coronary angiography and a 31% increase in the time from first medical contact to coronary revascularization. Gluckman et al. (33) evaluated in-hospital outcomes in 15,000 patients admitted for AMI at PSJH by dividing them into three periods: before COVID-19 (from December 30, 2018 to February 22, 2020), the early period of the pandemic (from February 23, 2020 to March 28, 2020), and late period of the pandemic (from March 28, 2020 to May 16, 2020). Besides reporting a decrease in AMI hospitalization of 19%, the study found that patients with AMI had a 50% increased risk of in-hospital death during the late period of the pandemic, even after adjusting for patient characteristics. In particular, based on the PSJH all-cause in-hospital mortality risk model, the observed/expected ratio for mortality related to all AMIs (STEMI and NSTEMI) was significantly increased in both the early period (at 1.27) and the late period of the pandemic (at 1.23). Our initial clinical experience is consistent with these worrying data. Of note, since the first patient was diagnosed with COVID-19 in Italy on February 20, 2020, we observed a significantly worse outcome in patients with STEMI when compared with that of the same time in the previous year (10). Notably, although the two cohorts were similar in terms of age, rate of diabetes mellitus, and history of previous AMI, we observed a two-fold longer time from symptom onset to hospital presentation (7.5 vs. 3.1 h) and a three-fold higher rate of cardiogenic shock (21 vs. 9%) and in-hospital cardiac mortality (19 vs. 5%) during the COVID-19 outbreak when compared with the same period of 2019. Similar figures have also been reported by other registries across countries (Figure 1). Thus, despite the limited time of these observations, initial reports indicate increased mortality in patients with AMI during the pandemic. However, the mechanisms underlying the worse short-term outcome cannot be deduced from these experiences. Yet, the significant delay in hospital presentation of patients with STEMI reported during COVID-19 may have resulted in a higher rate of mechanical complications and, consequently, in-hospital mortality. Of note, the long delay in the management of patients with STEMI was observed since the very first COVID-19 outbreak in Far East countries. A single-center study from Hong Kong that includes seven consecutive patients requiring primary PCI for STEMI during COVID-19 in January 2020 found longer median times from symptom onset to myocardial reperfusion when compared with the previous year (318 vs. 82 min) (47).

Besides the fear of infection, the late presentation of patients with AMI during the pandemic may also be related to delays in the field, with a longer response time of the emergency medical services due to safety precautions and changes in standard procedures. In-hospital delays may also be playing a role as evaluation in the emergency department and treatment in the catheterization laboratory entail lengthy procedures due to patient triage and donning of personal protective equipment (25, 40). Further work is needed to determine what factors contributed most to the decreased and delayed AMI presentation and increased mortality. In particular, a recent study reviewed all available information on the incidence of STEMI hospitalizations during the COVID-19 pandemic, worldwide, focusing on the possible factors underlying discrepant results (48). This study confirmed that during the first peak of the COVID-19 pandemic, there has been a significant decrease in STEMI hospitalizations worldwide. However, the magnitude of decrease was of a lesser extent than initially described. Most importantly, through a meta-analytical approach of a significant number of reports that includes >100,000 cases from 57 countries, and systematic assessment of various health-related metrics, substantial differences emerged among studies and countries, probably due to different functioning of hospital services and different hub-and-spoke approaches to STEMI, along with adequate public information during the pandemic (48). As different phases of the COVID-19 pandemic took shape, other investigations have been published to update the epidemiological picture of AMI, both at a national and at a global level. Indeed, a recent Canadian report assessed the changes in emergency department visit volume, care processes, and outcomes for stroke and AMI in the population of Ontario (49). It reported a reduction of 25–40% in emergency department visits for both acute diseases during the initial phase of the pandemic, with a subsequent compensatory increase in the late reopening phase and without a new drop during the second spread, starting in Ontario in September 2020 (49). Conversely, an English analysis comparing the daily incidence of hospital admission with AMI for the pre-COVID-19 period (November 2018 to March 2020) with that of the first and second UK lockdown found the second decline in admissions (by 34%) from the beginning of October 2020 up to November 2020, compared with the pre-COVID-19 period, despite an initial recovery in June 2020 (50).

In conclusion, regardless of the epidemiology of AMI during the COVID-19 outbreak and pandemic, there must be continued efforts through media attention on heart disease and public information to reduce patient fear to go to the hospital, emphasizing the importance of early recognition, and prompt treatment of AMI to ensure that COVID-19 management is no longer at the expense of this time-dependent disease. In this regard, the collateral damage of COVID-19 should not be ignored. Indeed, four different waves of the pandemic have been identified, involving different types of health impacts. After the first wave of immediate response to COVID-19, especially in terms of intensive care unit bed availability, a second non-COVID-19 wave of other urgent health conditions was neglected in the first one and a third wave defined by the result of interrupted care of chronic conditions have been clearly highlighted. Thus, these two waves not directly associated with the infection may have a negative impact on cardiovascular diseases during the following years, with an unprecedented increase in the prevalence of post-ischemic cardiomyopathy and heart failure. Finally, a fourth wave associated with the psychological trauma and economic injury caused by the pandemic can significantly affect the population in the future (51).

## The AMI Network During the COVID-19 Outbreak and Pandemic

A relevant indirect consequence of the COVID-19 pandemic is the adverse impact on the efficacy and effectiveness of the network organization required to offer primary patients from PCI to STEMI (and patients from PCI to AMI in general), with the appropriate standards of care, within appropriate time frames, and with dedicated preventive and containment measures against COVID-19 infection. Lombardy is the most densely populated region in Italy, with ~10 million inhabitants. With regard to the STEMI network, the healthcare system is divided into 8 areas, with an overall availability of 55 catheterization laboratories performing 24/7 primary PCI, and with a well-established STEMI network. However, during the COVID-19 outbreak, most hospitals underwent a sudden and radical transformation: all deferrable cardiac surgical and interventional procedures were delayed, the number of ICU capacity was exponentially increased, and most departments, such as ICCU, were converted to COVID-19 units (52). Notably, this disruptive effect on cardiovascular disease services has been common to many countries, as confirmed by an ESC survey, in which about 50% of the respondents reported that their cardiovascular wards or departments had undergone a logistical restructuring due to the pandemic (53).

To face the COVID-19 emergency, on March 8, 2020, the Government of Lombardy and local health authorities requested to centralize the regional treatment of cardiovascular time-dependent emergencies in a limited number of centers. Thus, a centralization model based on “macro-hubs” was developed for the treatment of STEMI. One or two macro-hubs were identified in each of the eight areas of the region, according to the estimated transportation time, geographical features, and capacity to admit all the potential patients (52). The following requirements were considered to become a macro-hub: to perform primary PCI to all-incoming STEMI on a 24/7 basis, to guarantee a primary PCI team available in hospital 24/7 and not on-call, and to provide dedicated and separated pathways for STEMI patients with suspected/diagnosed COVID-19 disease from triage, through catheterization laboratory and to isolated ICCU to reduce cross-infection risk. Thus, 13 macro-hubs were identified, with a 63% reduction in the number of the original pre-pandemic hubs. This model of STEMI centralization was established to keep the regional healthcare system from being overwhelmed, and to guarantee, at the same time, standard levels of care to patients with AMI (52). Not only the regional AMI network was modified to face the COVID-19 pandemic but also the in-hospital AMI pathways were changed accordingly. In our hospital, one of the 13 identified macro-hubs, we rapidly developed a local protocol for triage and management of patients with AMI (9). In particular, we attempted to identify a customized pathway to allocate patients to the appropriate hospital ward treat them according to the type and severity of AMI, and to the potential concomitant risk of infection. In patients presenting with STEMI at the emergency department or referred from spoke hospitals, conservative care was not considered an option, and they were immediately transferred to the catheterization laboratory for primary PCI. In particular, the interventional procedure was performed in a catheterization laboratory dedicated to emergencies of potentially infected patients, in whom there was not time to wait for the polymerase chain reaction result of the naso-pharyngeal swab. Patients with a high-risk NSTEMI, as defined by the presence of hemodynamic and/or electrical instability, recurrent or ongoing chest pain refractory to medical treatment, and/or relevant ST-T wave changes, followed the STEMI protocol. Conversely, patients with a low-intermediate risk NSTEMI were evaluated in the emergency department in a dedicated and monitored area and underwent naso-pharyngeal swab immediately after admission. In the case of positive swabs and clinical stability, PCI was deferred. If PCI was clinically indicated, it was performed in a catheterization laboratory dedicated to SARS-CoV-2-positive patients. All patients with AMI, regardless of the treatment modalities, were admitted to different wards according to their naso-pharyngeal swab results. The use of this in-hospital pathway focusing on patients with AMI was implemented in our hospital a few weeks after the beginning of the COVID-19 outbreak. Since June 2020, we have had a new device for rapid analysis of the naso-pharyngeal swab, with results being available within 20 min. This allowed to quickly allocate patients to the proper catheterization laboratory and monitored ward according to the presence or absence of SARS-CoV-2, greatly simplifying their in-hospital pathway (9).

Although firm conclusions on the safety and efficacy of the Lombardy centralization model for AMI management cannot be drawn now, initial experience has been reported in a registry (40). From February 21 to May 7, 2020, 953 patients with AMI were treated. The clinical presentation was STEMI in 58% of the cases and 98% of all patients received coronary angiography, followed by PCI in 84% of the cases. About half of the patients were transported to a macro-hub by the emergency medical service, while a fourth was transferred from the spoke centers. The median time since first medical contact to angiography was 79 min for STEMI and 1,262 min for NSTEMI. Eleven percent of study patients presented a concomitant SARS-CoV-2 infection with pneumonia in 60% of them. Interestingly, STEMI was the clinical presentation in most of these cases, a higher rate compared to that of COVID-19-negative patients (75 vs. 56%). Coronary angiography was performed in 98% of overall patients with COVID-19 and 80% of them underwent PCI. No patient with STEMI was treated with fibrinolysis (40). Thus, during the 2 months with the highest daily increase of COVID-19 cases in Lombardy, nearly all patients received a timely coronary angiography and their treatment time since first medical contact was in line with guidelines recommendations. Although being a preliminary experience, the redefinition of AMI network based on macro-hubs seems to allow physicians to continue with timely AMI management, while reserving a high number of ICU beds for the pandemic. Preliminary data, comparing the second spread (November 2020 to January 2021) to the first one of the pandemic in the same Macro-Hubs in Lombardy, revealed no significant differences in clinical presentation and in the time from symptom onset to first medical contact, with a significant reduction in mortality and time to treatment during the second wave, further supporting the crucial role of centralization model applied in Lombardy (Ferlini et al., submitted).

During the COVID-19 pandemic, the management of patients with acute coronary syndromes has changed worldwide, and several protocols have been developed to guarantee the best treatment while minimizing the virus spread. Chinese physicians of the Sichuan Provincial People's hospital coping with the first wave of the pandemic proposed fibrinolysis as the treatment of choice for stable COVID-19 positive patients with STEMI. Elective PCI was then only considered after patient recovery from COVID-19 pneumonia, regardless of whether the patient was evaluated at a primary PCI center or not (54). Conversely, a primary-PCI strategy for COVID-19 patients with STEMI was the recommended one in a Singapore experience. Moreover, in that center, prophylactic early elective intubation was performed in cases characterized by a likely respiratory deterioration. This approach allowed to avoid emergency intubation in such frail patients and to reduce the risk of catheterization laboratory staff exposure (55).

Besides these locally developed coping strategies, the main scientific societies have been very active in assisting clinical and interventional cardiologists. A Consensus Statement from the Society for Cardiovascular Angiography and Interventions (SCAI), the American College of Cardiology (ACC), and the American College of Emergency Physicians (ACEP) was published in April 2020 to provide a systematic approach for the care of patients with AMI during the COVID-19 pandemic (56). According to this document, in the case of a STEMI seen at a primary PCI center, the treatment slightly differed whether the patient was a COVID-19-positive/probable or possible (based on an ultra-rapid COVID-19 test). A COVID-19 positive/probable patient with classic clinical symptoms and ECG findings was considered for ultrasound evaluation of cardiac function to assess regional wall motion abnormalities consistent with the ECG findings before undergoing primary PCI. On the contrary, COVID-19 possible patients with classic clinical presentation and ECG finding consistent with a STEMI proceeded directly to primary PCI. In the case of a diagnosis of STEMI in non-PCI-capable hospitals, the primary PCI remained the standard of care for patients in whom reperfusion within 120 min of first medical contact at referral hospital was feasible. Only patients who could not be rapidly moved to the primary PCI center underwent fibrinolysis before transfer. Finally, as regards to patients with NSTEMI, COVID-19-positive or probable patients were initially managed medically and only taken for urgent coronary angiography and possible PCI in the presence of high-risk clinical features. Finally, a document by the ESC was published to help physicians dealing with cardiovascular disease during the COVID-19 pandemic. As in other protocols, a distinction between NSTEMI and STEMI was made. While patients with NSTEMI are suggested to be managed according to risk stratification (very high risk—treated as patients with STEMI, high risk, intermediate risk, and low risk), this indication does not apply to patients with STEMI, to guarantee timely reperfusion. According to the ESC document, all patients with STEMI should be managed as COVID-19 positive, in the absence of previous SARS-CoV-2 testing, to ensure the safety of healthcare personnel (57, 58).

## Lessons Learned and Conclusion

The COVID-19 pandemic took the healthcare system worldwide by surprise and “distracted” physician's attention from the management of cardiovascular diseases, particularly time-dependent emergencies, with critical repercussions on the effectiveness of life-saving treatments and patient prognosis. After an initial shock, physicians realized that timely management of cardiac emergencies with appropriate standards of care should be ensured even during major unpredictable events. This can be achieved through a timely adoption of countermeasures against this unprecedented and dramatic emergency aimed at preventing large and long-standing health and social impact. In particular, health authorities should implement protocols that may provide a response to the index emergency and, at the same time, guarantee the best treatment strategy for AMI, based on prompt changes in the hub and spoke interplay. The delay in treatment delivery has also been a matter of serious concern raised during the COVID-19 pandemic, limiting the effectiveness of life-saving therapies for AMI. Indeed, patients have been reluctant to go to the hospital due to the fear of COVID-19, with many patients with AMI not seeking care at all or only late in the course of the acute event. This has contributed to increase the death toll beyond levels directly associated with SARS-CoV-2 infection. Although many questions remain unanswered and further evidence should be collected, we believe that every effort should be made by scientific societies, health authorities, and public media to convince patients not to delay life-saving treatments, even during dynamic crises.

In conclusion, in case the health situation returns to critical emergency levels, the experience gained during the COVID-19 pandemic should be an instructive lesson to help us be better prepared and provide appropriate guidance based on evidence on how to maintain optimal AMI management, even when the healthcare systems are under extreme strain.

## Author Contributions

GM, NC, and AB: concept and design. OT and JC: drafting the manuscript. GM and AB: study supervision. All authors contributed to the article and approved the submitted version.

## Conflict of Interest

The authors declare that the research was conducted in the absence of any commercial or financial relationships that could be construed as a potential conflict of interest.

## Publisher's Note

All claims expressed in this article are solely those of the authors and do not necessarily represent those of their affiliated organizations, or those of the publisher, the editors and the reviewers. Any product that may be evaluated in this article, or claim that may be made by its manufacturer, is not guaranteed or endorsed by the publisher.
